# Porogen Concentration Effect on the Pore Structure and Properties Evolution of Polymer Monolith Based on Oligocarbonate Dimethacrylate OCM-2

**DOI:** 10.3390/ma16083177

**Published:** 2023-04-18

**Authors:** Roman S. Kovylin, Vladimir V. Yudin, Margarita P. Shurygina, Victor B. Fedoseev, Sergey A. Chesnokov, Igor L. Fedushkin, Alexandr V. Piskunov

**Affiliations:** G.A. Razuvaev Institute of Organometallic Chemistry of Russian Academy of Sciences, Tropinina 49, Nizhny Novgorod 603950, Russia; yudin@iomc.ras.ru (V.V.Y.);

**Keywords:** photopolymerization, porous polymers, pore structure

## Abstract

Porous polymer monolith materials of 2-mm thickness were obtained by visible light-induced radical polymerization of oligocarbonate dimethacrylate (OCM-2) in the presence of 1-butanol (10 to 70 wt %) as a porogenic additive. The pore characteristics and morphology of polymers were studied by mercury intrusion porosimetry and scanning electron microscopy. Monolithic polymers with both open and closed pores up to 100 nm in size are formed when the alcohol content in the initial composition is up to 20 wt %. The pore structure in such materials is a system of holes in the bulk of the polymer (hole-type pores). Open interconnected pores with a specific volume up to 2.22 cm^3^/g and modal pore size up to 10 microns are formed in the volume of the polymer with 1-butanol content of more than 30 wt %. Such porous monoliths are a structure of covalently bonded polymer globules (interparticle-type pores). The free space between the globules represents a system of open interconnected pores. In the transition region of 1-butanol concentrations (from 20 to 30 wt %), areas with both structures and intermediate frameworks, as well as honeycomb structures of polymer globules connected by bridges, are fixed on the polymer surface. It was found that the transition from one type of pore system to another is accompanied by a sharp change in the strength characteristics of the polymer. Approximation of experimental data using the sigmoid function made it possible to determine the concentration of the porogenic agent in the vicinity of which the percolation threshold is observed.

## 1. Introduction

The synthesis of porous polymers with a given pore structure and morphology is a topical problem of modern polymer chemistry. The main application areas of porous polymers are high-performance electronics [1,2], gas storage [3,4], tissue engineering [5,6], the creation of separating materials [7,8], adsorbents for various purposes [9,10], devices for controlled drug delivery [11], as well as reagents and catalysts on a polymer carrier [12]. Porous polymers with a tunable open-pore structure can be obtained by nanolithography [13]. However, the low performance of the method does not yet allow its use for practical purposes. Free radical polymerization is one of the most common methods to obtain meso- and macroporous polymers [14]. Its essence is a curing of a homogeneous mixture monomer, and/or a crosslinker (for example, bifunctional di(meth)acrylate oligoester) and a porogenic agent. In this case, it is essential that the porogenic agent gives a homogeneous mixture with the initial components and is a thermodynamically poor solvent for the synthesized polymer. Formation of a porous structure occurs due to the phase separation of the resulting polymer and porogenic agent. The efficiency of this process is determined by the thermodynamic compatibility of the polymer and the porogenic agent. The value of thermodynamic affinity can be estimated based on the Hildebrand solubility parameters (δ) for the polymer and porogenic agent [15,16]. The greater the difference in solubility parameters of the polymer (δ_pol_) and porogenic agent (δ_solv_), the lower their thermodynamic affinity and the more expressed the effect of the phase separation.

Despite the widespread use of free radical polymerization, there are few publications devoted to systematic experimental studies on the effect of reagents nature and its concentration on the efficiency of pore formation. The compositions based on dimethacrylates (ethylene glycol dimethacrylate [17,18,19,20] and glycerol dimethacrylate [15,21]) are widely used. Aliphatic alcohols at a concentration of more than 50 vol % are often added as porogens [15,18,19,20,21]. It has been shown that polymerization of ethylene glycol dimethacrylate in the presence of aliphatic alcohols [22] and oligocarbonate dimethacrylate OCM-2 in the presence of dialkylphthalates [23] or a mixture of aliphatic alcohols [24] leads to the formation of biocompatible non-cytotoxic porous polymer monoliths when the condition Δδ = |δ_pol_ − δ_solv_| > 3 MPa^1/2^ is fulfilled. It was proved by sorption and gravimetry methods that, despite the thermodynamic incompatibility of crosslinked polyOCM-2 and dialkyl phthalates, the process of pore formation starts only with a certain concentration of the porogen in the initial composition [23]. Clearly, the appearance of a porous structure in the bulk of the polymer should lead to a change in both the morphology of the polymer and its properties. Such changes in a specific polymerizing system dimethacrylate–porogenic agent are determined by the content of the porogenic agent.

Porous polyOCM-2 samples were synthesized by free radical photopolymerization under visible light irradiation. In comparison with the thermal initiation of reaction, the photopolymerization allows us to (i) reduce the synthesis time, (ii) carry out the process in a wide temperature range, and (iii) use low-boiling organic liquids as porogens [25]. UV-sensitive photopolymerizable compositions (PPC) are mainly used for this [26,27]. However, due to the fact that the composition components and the resulting polymer structure absorb and scatter the initiating irradiation, the UV polymerization rate abruptly decreases in the layer depth and the photopolymerization process occurs in thin layers of PPC only [28]. This limits the possibility of studying the polymer structure in the inner regions of thick layers. To overcome this drawback, we used initiation systems that are sensitive to visible irradiation because they allow us to obtain porous polymer blocks with a thickness of 4 mm, which have uniform properties [23]. The polymers were studied by electron spectroscopy, mercury intrusion porosimetry (MIP), and scanning electron microscopy (SEM). Their physical and mechanical properties were also determined.

In the past two decades, the number of studies concerning scaffold development and tissue engineering has increased exponentially [29]. Porous biocompatible natural and synthetic polymer materials have taken leading positions among the materials which are suitable for the designing of customized implants. Their properties can be varied and adjusted to a specific task. In this regard, it is important to study the effect of porogenic agent concentration on the evolution of pore structure, morphology, and the properties of biocompatible polymer monoliths. Biocompatible non-cytotoxic polymer based on oligocarbonate dimethacrylate OCM-2 [23,24] is a promising material for these purposes. It is known that the polyOCM-2 swelling equilibrium constant in 1-butanol is close to zero [23], which indicates the thermodynamic incompatibility of polyOCM-2 and 1-butanol. This makes it possible to analyze the polymers obtained by the polymerization of OCM-2 in the presence of 1-butanol in a wide range of porogenic agent concentrations (from 10 to 70 wt %). In this paper, we report the influence of 1-butanol concentration on the pore structure and morphology of the porous monoliths derived from OCM-2. To do this, in the first stage of the work, studies of the pore structure of the obtained polymers by mercury porosimetry and scanning electron microscopy were carried out. The second stage consisted in searching for the relationship between the strength characteristics and optical properties of porous polymers depending on the pore structure of the material. Understanding the evolution of the pore structure with a change in the concentration of the porogenic agent will facilitate the selection of polymer synthesis conditions depending on the target values of porosity, pore structure and their size distribution.

## 2. Materials and Methods

### 2.1. Materials

**Figure 1:**
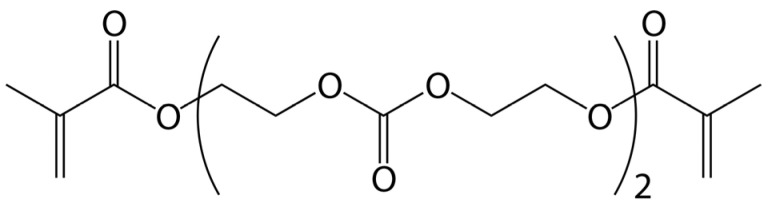
Chemical structure of OCM-2 oligomer.

α,ω-Bis(methacryloyloxyethyleneoxycarbonyloxy)ethyleneoxy-ethylene (OCM-2, Figure 1) (99.9%, ReperNN Ltd., Nizhny Novgorod, Russia), isopropyl alcohol (99.5%, Aldrich, USA) and 1-butanol (1-BuOH) (99.5%, Aldrich, USA) were used as purchased. The photopolymerizable composition was prepared by dissolving the initiator in a mixture of OCM-2 with the addition of porogenic agent 1-butanol. The concentrations of the alcohol ranged from 10 to 70 wt %. Initiation was performed by visible light irradiation of the photosensitive system comprising 3,6-di-tert-butylbenzoquinone-1,2 (99%, Sigma-Aldrich, USA) (0.05 wt %) and N,N-dimethylethanolamine (99.5%, Sigma-Aldrich, USA) (1.0 wt %) [30,31].

### 2.2. Synthesis of Porous Polymers

**Figure 2:**
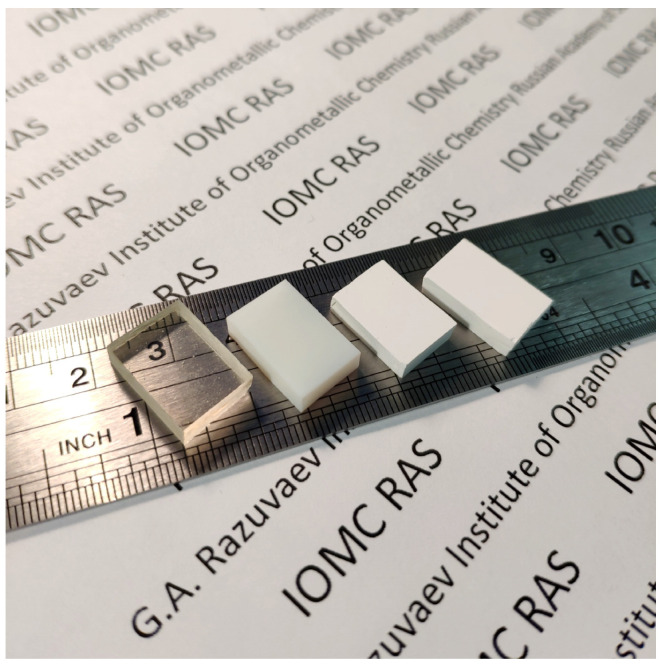
Photograph of 4 mm thickness porous polymer obtained from various compositions of OCM-2-1-butanol.

Porous polymer samples (Figure 2) were prepared according to the previously developed method [30]. The photopolymerizable composition was placed in a mold formed by two flat silicate glasses with a 2.0- or 4.0-mm gasket. The glasses were not treated with any modifying liquids. The exposure started after 1 min after pouring the composition into a mold. A Philips UHP halogen lamp (400–750 nm, 190 W) was a source of the initiating irradiation. An emission spectrum of the lamp has been studied previously [23]. The composition was irradiated for 1 h at an illumination of I = 50 kLx, resulting in the cross-linked poly-OCM-2. After removal from the mold, samples were washed with isopropyl alcohol in a Soxhlet apparatus at least 50 times. The alcohol was removed from the polymeric monolith by evacuation at 60 °C for 24 h. Non-porous polymer glasses were obtained without the addition of a porogen by the same procedure with the difference that the samples after exposure were not washed with isopropyl alcohol.

### 2.3. Measurement

The surfaces of the polymer samples were investigated by SEM using Regulus SU8100 (Hitachi, Japan). The porous properties (modal pore diameter D_mod_, specific surface area S_sp_, porosity ε, total pore volume V_total_) of poly-OCM-2 monoliths were determined by mercury intrusion porosimetry using a Pascal 140 and 440 (Thermo Fisher Scientic, Rodano, Italy) mercury porosimeter. Prior to the measurements, the polymer monoliths were cut into small pieces (100–200 mg) using a razor blade. The compressive elastic modulus (E_C_) and compressive breaking strength (σ_B_) of porous poly-OCM-2 monoliths were determined according to ISO 604:2002 (https://cdn.standards.iteh.ai/samples/31261/7596c06eb39540f3ae2f1abcdae4fb79/ISO-604-2002.pdf, https://www.iso.org/standard/31261.html). The samples were cut into 10 × 10 × 4 mm blocks. Furthermore, the blocks were compressed using an AGX-V 50kND (Shimadzu, Kyoto, Japan) universal testing machine at a strain rate of 0.5 mm min^−1^ on 10 × 10 × 8 mm samples (8 mm thickness was provided by applying two polymer specimens). The compressive modulus was calculated from the slope of the linear region and the compressive strength was identified, after correcting for zero strain, as the stress at 10% strain. Reported E_C_ and σ_B_ data were averaged for five specimens for each poly-OCM-2 composition. Electronic absorption spectra were obtained on an SF-56 spectrophotometer (LOMO, Saint Petersburg, Russia).

## 3. Results

A series of 14 porous polymer monoliths based on polyOCM-2 was obtained by visible light photopolymerization. The alcohol content in the initial photopolymerizable compositions varied from 0 to 70 wt %. Due to a very strong change in the position and intensity of the maxima on the pore size distribution curves of the obtained polymers, they were divided into three groups depending on the content of the 1-butanol: (i) low concentrations—from 0 to 20 wt %; (ii) average concentrations—from 25 to 50 wt %; (iii) high concentrations—60 and 70 wt %. The pore characteristics (modal pore size D_mod_, porosity ε, specific surface area S_sp_, total pore volume V_total_) of polyOCM-2 samples depending on the content of the porogen 1-butanol are shown in Table 1. The density value of polyOCM-2 equal to 1.21 g/cm^3^ [23] was used to calculate the volumetric concentration of the 1-butanol.

### 3.1. Low Concentrations (from 0 to 20 wt %) of the Porogen 1-Butanol

**Figure 3:**
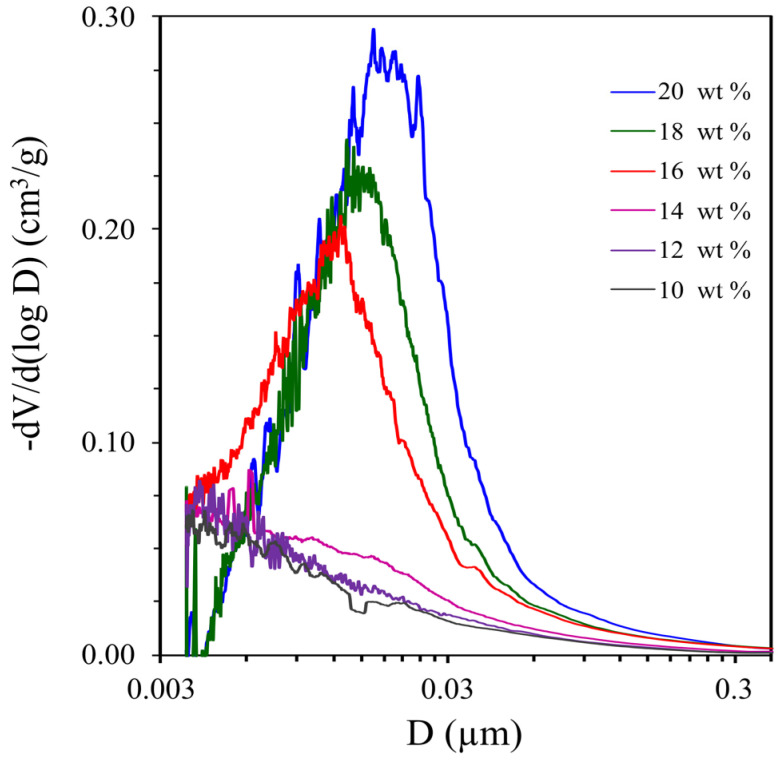
The pore size distribution of poly-OCM-2 monoliths (M2-M7) obtained from OCM-2 in the presence of 1-butanol (10–20 wt %).

Porous polymer monoliths M1–M7, depending on the concentration of the porogen, are strong transparent, turbid or white materials. Pore size distributions (Figure 3) for the samples M1–M7 were determined by the mercury porosimetry method. The values of the porosity, specific surface area and pore volume in these samples are shown in Table 1. A slight rise corresponding to a modal pore diameter of about 4 nm (Figure 3, curves 1, 2) in the M1 and M2 polymers pore size distribution is observed at an alcohol concentration of 10 and 12 wt %. An increase in the alcohol concentration to 14 wt % leads to an insignificant increase in the modal pore diameter to 6.2 nm (Figure 3, curve 3). The form of the polymer pore size distribution changes at 16 wt % alcohol (Figure 3, curve 4). Pores up to 60 nm in size appear and the largest contribution to the total pore volume of the polymer is made by pores with a pore diameter of 12.7 nm. According to Table 1, the values of porosity and the specific surface area of porous polymers double with an increase in the alcohol content from 14 to 16 wt %. The total pore volume of polymers increases from 0.05 to 0.18 cm^3^/g, and the modal pore diameter increases to 19.7 nm with an increase in the alcohol concentration to 20 wt % (Table 1).

**Figure 4:**
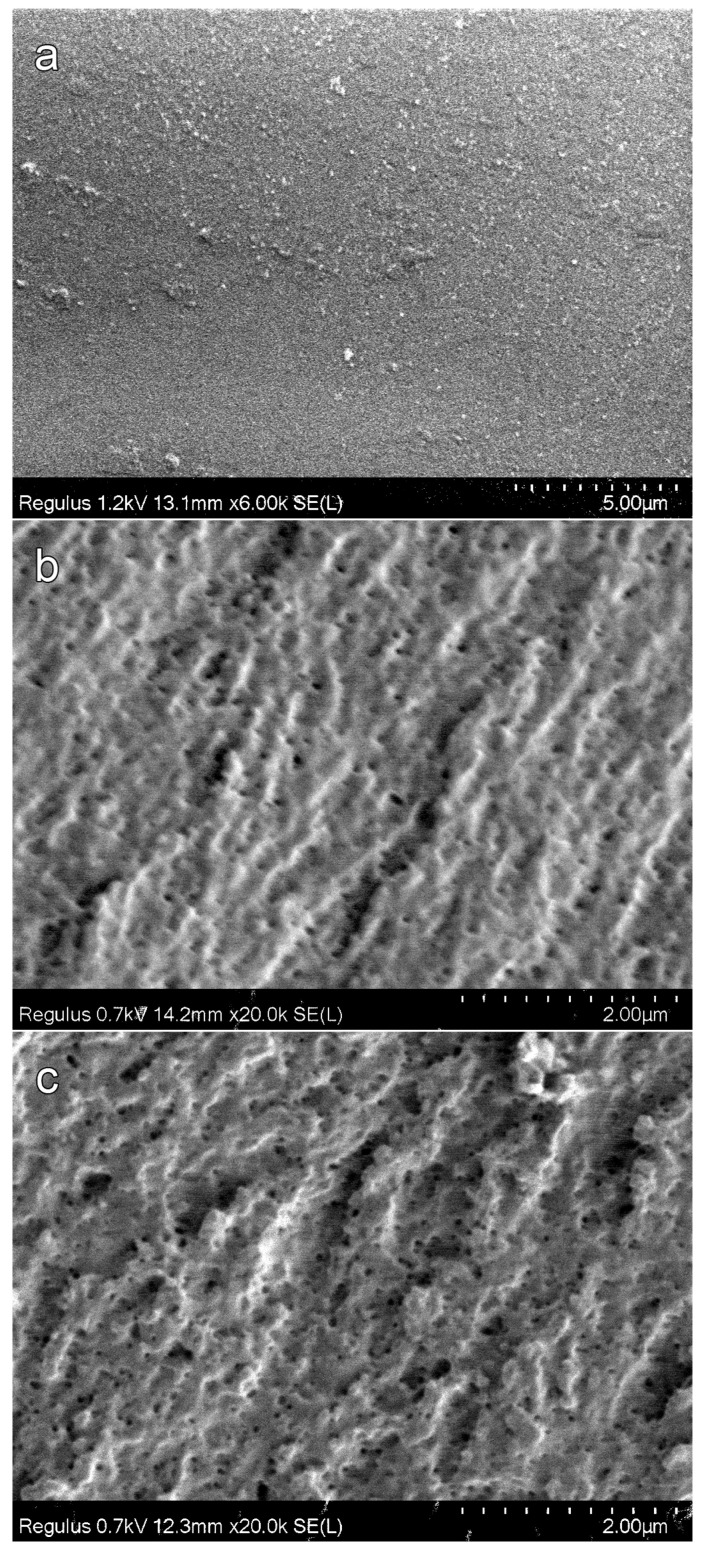

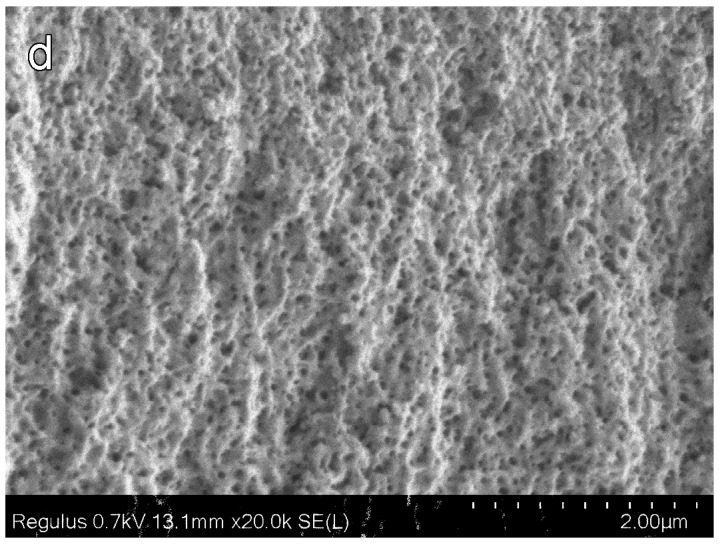
SEM micrographs cleavage surfaces of porous poly-OCM-2 monoliths prepared with various 1-butanol concentrations: (**a**) 0% (M1); (**b**) 12% (M3); (**c**) 16% (M5); (**d**) 18% (M6).

Figure 4 shows the SEM images for the porous polyOCM-2 monoliths (M1–M6) with different concentrations (0–20 wt %) of the porogen 1-butanol. The cleavage surface of polymer M1 obtained without porogenic alcohol has no pores (Figure 4a). Disordered holes less than 100 nm in size are observed on the cleavage surface of polymer M3 (12 wt % alcohol content, Figure 4b). The number of such holes increases with an increase in the concentration of 1-butanol. The number and size of pores with a further increase in the 1-butanol content in the PPC from 16 (M5, Figure 4c) to 18 wt % (M6, Figure 4d) significantly increase. Most of the holes become about 100 nm in size. Voids of an irregular shape up to 500 nm in size are observed on the polymer cleavage surface (Figure 4d).

### 3.2. Average Concentrations (from 25 to 50 wt %) of the Porogen 1-Butanol

**Figure 5:**
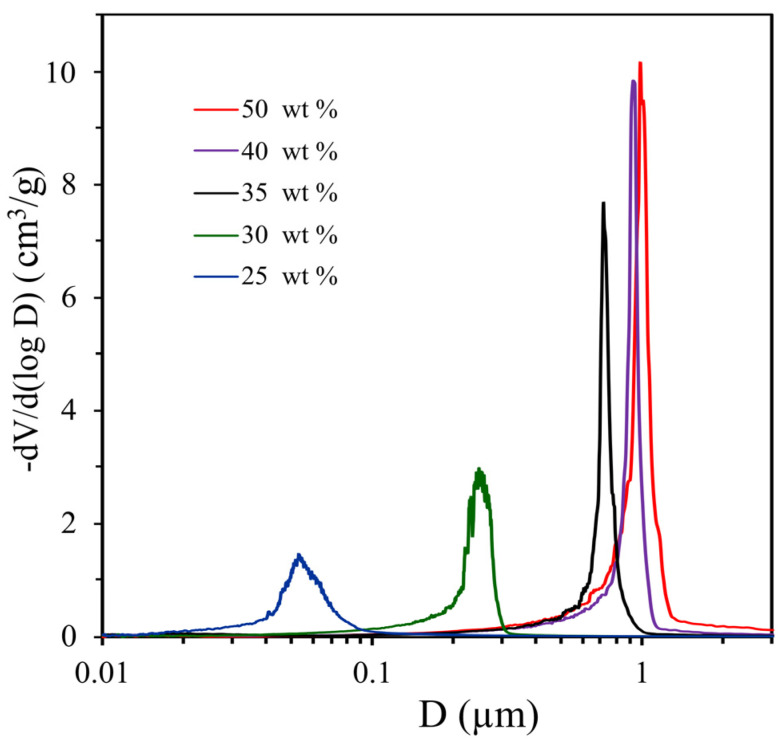
The pore size distribution of poly-OCM-2 (M8–M12) obtained from OCM-2 in the presence of 1-butanol (25–50 wt %).

Figure 5 shows the pore size distribution of polyOKM-2 (M8–M12) samples obtained with 1-butanol concentration from 25 to 50 wt %. Its porous properties are presented in Table 1. With an increase in 1-butanol concentration in FPC from 25 to 40 wt %, the values of the average size and specific volume of pores sequentially increase from 53 to 980 nm and from 0.34 to 1.11 cm^3^/g, respectively. As expected, an increase in the pore size leads to a consistent decrease in the specific surface areas of porous polymer monoliths from 36.3 to 15.9 m^2^/g for M8 and M12, respectively (Table 1).

**Figure 6:**
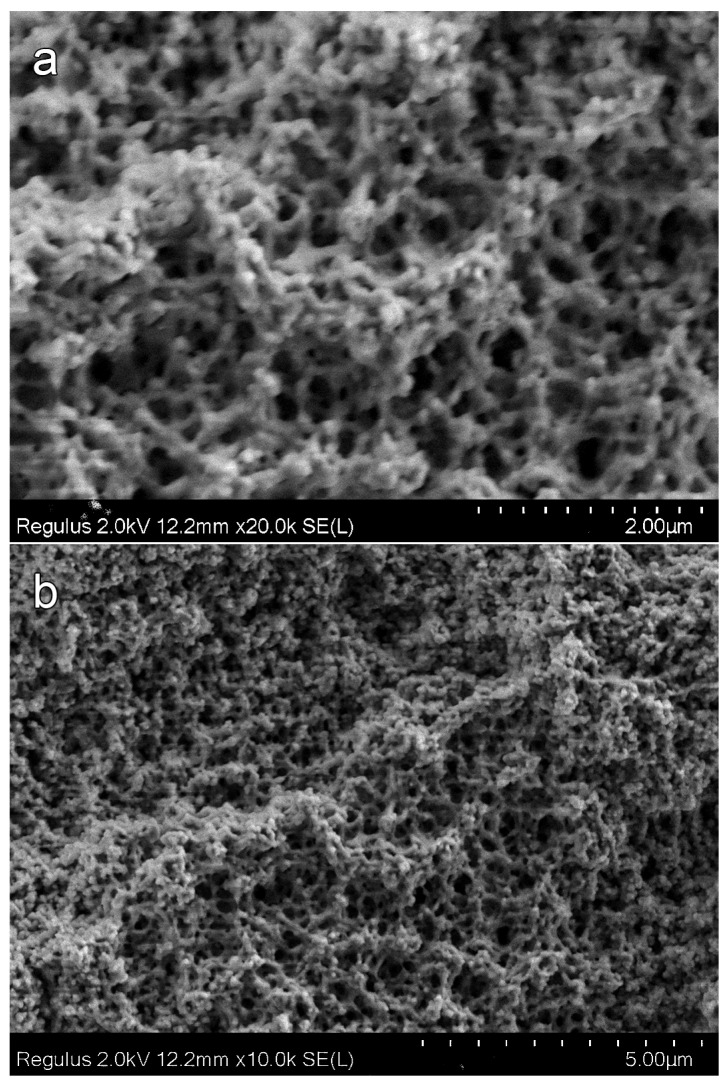

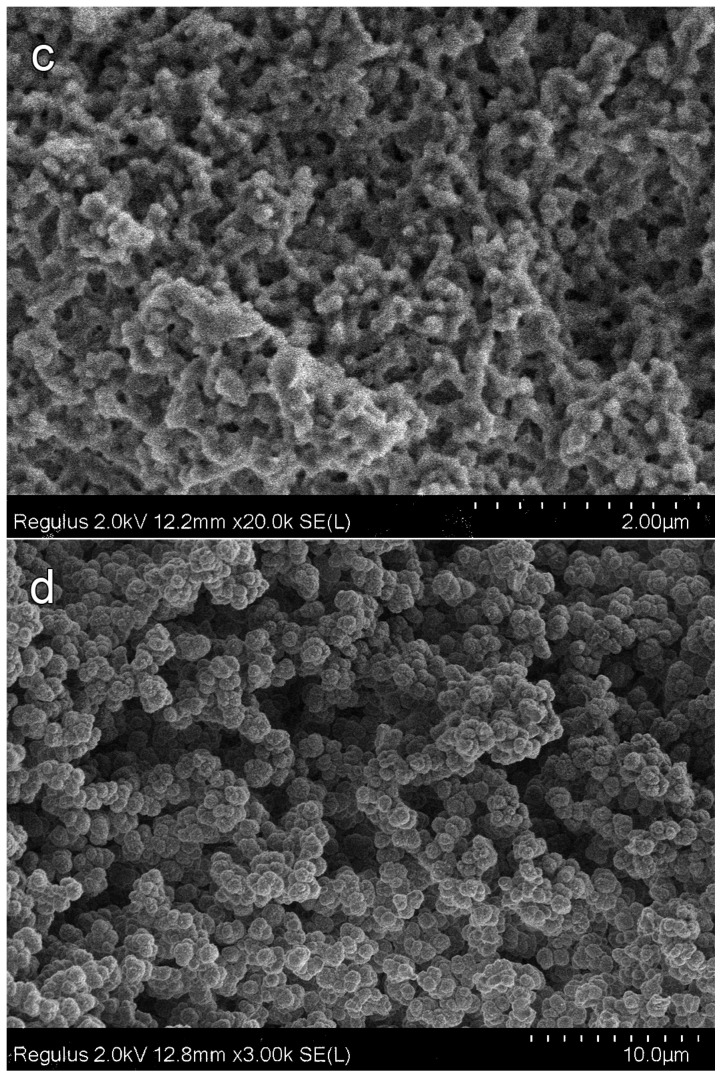
SEM micrographs cleavage surfaces of porous poly-OCM-2 monoliths prepared with various 1-butanol concentrations: (**a**,**b**) 25% (M8); (**c**) 30% (M9); (**d**) 50% (M12).

Figure 6 presents M8–M12 polymers cleavage surfaces SEM micrographs. SEM micrographs of the M8 polymer cleavage surface (25 wt % alcohol content, Figure 6a) show that the pore structure of this polymer is much more complicated than that of samples M1–M7 with pores in the form of holes. Polymer M8 includes two types of areas with a different pore structure: (i) a hole-type pore and (ii) an interparticle-type pore. Hole-type pore areas predominantly consist of holes, but compared to the M1–M7 polymers discussed above, the holes completely cover the surface (Figure 6b). In the interparticle-type pore areas, the polymer forms globules with a size of 100–200 nm, which are connected to each other by cylindrical bridges. These areas with different pore structures pass into each other with the formation of three-dimensional frameworks. In a number of cases (Figure 6b), the structure of the voids is close to that of a honeycomb and is formed by bridging several (usually six) polymer globules up to 100 nm in size. SEM micrographs of M9 and M12 polymers cleavage surfaces are presented in Figure 6c,d. An increase in the 1-butanol content to 30 and then 50 wt % leads to the formation of polymers with only an interparticle-type pore structure. They are entirely formed from cross-linked poly OCM-2 agglomerates of 2–3 μm size. In addition to pores of ~1 μm in size, voids up to 10 μm in size are observed on polymer cleavage surfaces.

### 3.3. High Concentrations (60 and 70 wt %) of the Porogen 1-Butanol

**Figure 7:**
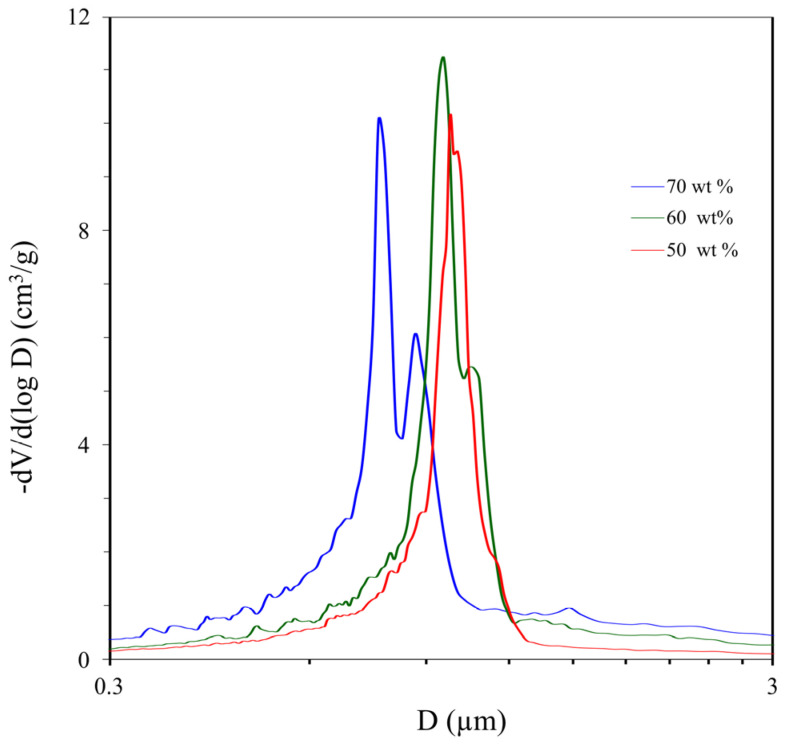
The pore size distribution of poly-OCM-2 (M12-M14) obtained from OCM-2 in the presence of 1-butanol (50–70 wt %).

Figure 7 shows the pore size distribution of polyOCM-2 samples (M12–M14) obtained in the presence of 50, 60 and 70 wt % 1-butanol. The pore size distribution of porous polymer M12 is shown again in Figure 7 as a benchmark. It can be seen that the modal pore diameter does not increase, as was the case for M2–M11 samples, but decreases. In the pore size distribution curves of M13 and M14 polymers, in contrast to the M12 sample, there is a second minor peak, and the proportion of pores larger than 1 μm in size is higher.

**Figure 8:**
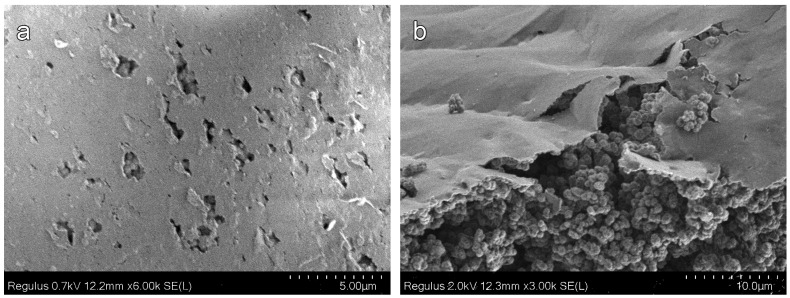
SEM micrographs of the film on the outer surfaces of porous monoliths: (**a**) M8 (25 wt % alcohol content) and (**b**) M13 (60 wt % alcohol content).

During photopolymerization in a mold of silicate glasses, a thin film is formed on both sides (light side and against light side) of the outer surface of the samples. Therefore, all the previously presented SEM images of porous polymers were obtained for sample cleavages. A film is clearly visible in micrographs of polymers M8 and M13 (25 and 60 wt % alcohol content). Figure 8 shows that the samples are covered with a ~1 μm thick film. In both cases, the pore structure of the polymer is visible under the film.

**Figure 9:**
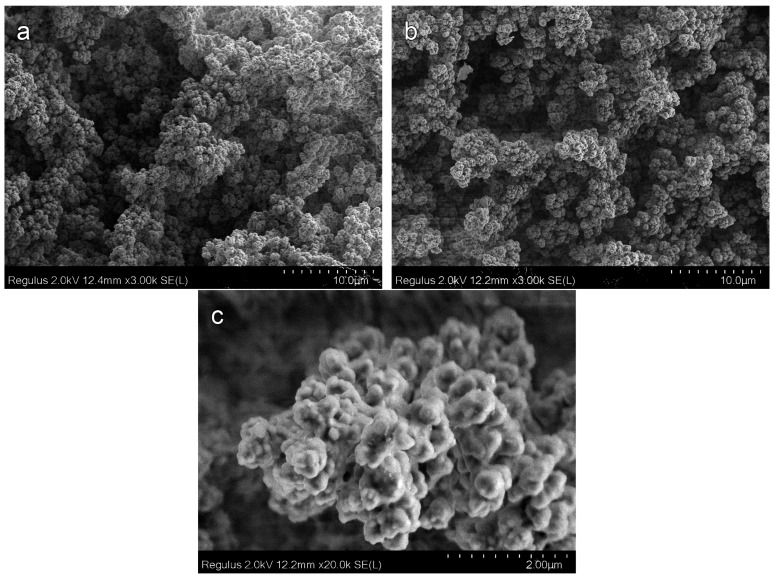
SEM micrographs cleavage surfaces (**a**) and outer surface of the sample side (**b**,**c**) of porous M14 (70 wt % alcohol content) monolith.

Figure 9 shows SEM micrographs of the outer surface and the cleavage surface of the polymer monolith M14 (70 wt % alcohol content). There is no film on the sample of the outer surface. The porous structures on the cleavage surface (Figure 9a) and the outer surface of the sample side (Figure 9b) are similar to each other. According to SEM data, the M14 polymer pore size is the maximum of the entire investigated series of polymers and reaches values of 10 μm or more. The pore surface is an agglomerate of spherical particles of 1–5 µm in size, outwardly similar to coral (Figure 9c). Continuous free space as a system of interconnected pores exists between the clusters of particles. A certain hierarchy of pores can be seen in the micrographs. The cavities of the minimum size in the contact areas of particles clusters have a size of ~1 μm. Polymer clusters form various-shaped walls of pores of 10 μm or more.

## 4. Discussion

### 4.1. MIP and SEM Data Comparison

Based on the mercury intrusion porosimetry data, the obtained porous polymers can be divided into two groups. The first is polymers made from compositions with a 1-butanol content of up to 20 wt %. They are characterized by a small modal pore size (4–20 nm). The porosity of these polymers (pore volume fraction) is less than the volume fraction of the porogen 1-butanol in the compositions from which they are synthesized. Polymer M2, obtained in the presence of 10 wt % (14 vol %) 1-butanol, has a difference between these values Δε = 7.6% (Table 1). This value is greater than the porosity of the polymer M2 (ε = 6.4%). Consequently, in polymer M2 fewer than half of the pores (46%) are open and the other pores are closed and inaccessible to mercury intrusion. Similar patterns are observed for M3 and M4 polymers obtained with 12 and 14 wt % (16.9 and 19.6 vol %, respectively) 1-butanol content. The fraction of closed pores in polymer M7 (Table 1, 1-butanol concentration 27 vol %, ε = 19%) decreases to 30% with an increasing alcohol concentration.

The fraction of closed pores in polymer M8 (25 wt % alcohol content, Table 1) sharply decreases to 3.6% (1-butanol concentration is 33.3 vol %, ε = 32.1%). Most of the pores in sample M8 become open. In polymers M9–M14 obtained at a 1-butanol concentration of more than 25 wt %, the proportion of closed pores also remains minimal (Table 1). These polymers are characterized by large average pore sizes—hundreds of nanometers. With an increase in 1-butanol concentration, the specific surface area of the polymers changes. It increases from 19.8 m^2^/g (M2) to 46.3 m^2^/g (M7), and then gradually decreases to values of about 20 m^2^/g. Thus, polymers from compositions with 30 wt % of 1-butanol and more constitute the second group of polymers. Polymer M8 has a transitional pore structure relative to the above two groups of polymers.

SEM micrographs confirm this division. The cleavage surface morphology of polymers changes drastically when passing from polymers of the first group—with pores in the form of a system of holes, to polymers of the second group—interconnected pores (Figure 4, Figure 6 and Figure 9). The cleavage surface of the M8 polymer combines both variants of structures.

Thus, despite the thermodynamic incompatibility of the polymer (polyOCM-2) and porogen (1-butanol), an increase in alcohol concentration in the composition does not lead to a proportional change in the morphology of the polymer. The polymerization process in the presence of a low porogen concentration leads to a microphase separation of the polymerizing mixture and displacement of the porogen into local areas that are not connected to each other. In such systems, predominantly closed pores are formed, as can be observed for the M2 polymer (10 wt % alcohol content). An increase in porogen concentration leads to a decrease in the closed pores fraction. When a certain porogen concentration is reached, a single system of interconnected open pores is formed. For the investigated photopolymerizable system 1-butanol—OCM-2, this effect occurs at an alcohol concentration of 25 wt %, which corresponds to 33.3 vol %. With a further increase in porogen concentration in the composition, the volume of pores and their size in the polymer increase, but the type of pore structure of the resulting polymer does not change.

### 4.2. Percolation Effect

**Figure 10:**
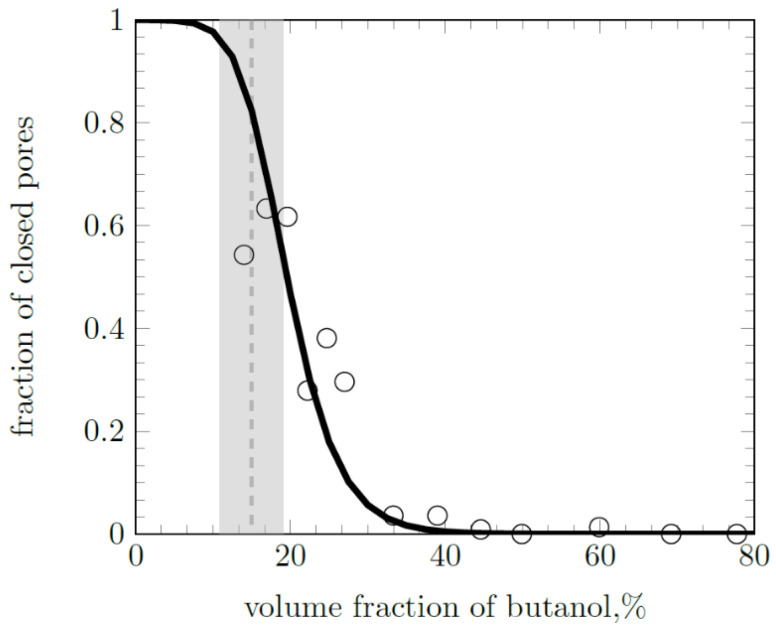
Fraction of closed pores depending on volume fraction of porogenic agent. The region of the percolation threshold is highlighted in gray.

Closed porosity values (Δε) of the obtained polymers are calculated and shown in Table 1. The obtained Δε values differ noticeably from the theoretical models used to describe porous materials in the theory of percolation [32]. This may be due to the strong influence of the polymerizable composition components surface activity on the pore structure formation. In this article, we propose a more flexible analytical model for assessing closed porosity based on sigmoid functions of the type:

(1) $${y(x)=1-(1+exp(-a*(x-b)))}^{-c},$$

where *a*, *b*, *c* are numerical parameters. Parameter b determines the region of the percolation threshold, parameters a and c change the shape (slope and curvature) of the curve. Closed pores predominate below the percolation threshold (1-butanol volume fraction < *b*). Closed porosity values (Δε) consistently decrease with an increase in the volume fraction of the porogenic agent. It is generally assumed that, below the threshold, percolation is completely suppressed [32,33]. In our case, the use of the sigmoid function *y*(*x*) determines the non-zero probability of percolation at the subthreshold values of the porogenic agent volume fraction. The fraction of closed pores (*ω*_closed_) values was determined as the ratio of the closed porosity value (Δε) to the volume fraction of the porogenic agent in the composition. Figure 10 shows the dependence of the closed pores fraction (*ω*_closed_) on the porogenic agent concentration. This dependence can be approximated by a sigmoid function (Figure 10, solid line) of the type:

(2) $${\omega_{closed}(\omega )=1-(1+exp(-25*(\omega -0.15)))}^{-2.5},$$

where *ω*—volume fraction of the porogenic agent. These data indicate the existence of a percolation threshold in the vicinity of a porogenic agent concentration of 15 vol %. The region of the percolation threshold is highlighted in gray. Comparable values of the percolation threshold were previously observed in polymer nanocomposites [33].

### 4.3. Changing the Light Transmission of polyOCM-2 Porous Monoliths

**Figure 11:**
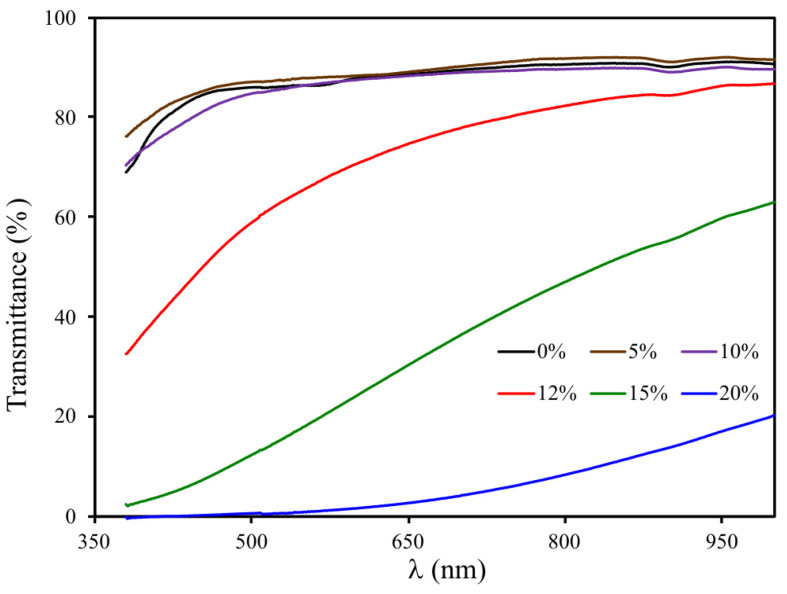
Transmission spectra of polyOCM-2 porous polymer monoliths depending on the content of the porogen 1-butanol (0–20 wt %).

A change in the morphology of a porous polymer is accompanied by a change in its “macro” properties. The transmission spectra of 2-mm-thick porous polymer monoliths obtained from OCM-2 at a concentration of 1-butanol 0–20 wt % are presented in Figure 11. It can be seen that the addition of 5 and 10 wt % 1-butanol to the PPC has a weak effect on the spectral characteristics of the porous polymer monolith. A further increase in the alcohol concentration by 1 and 2 wt % leads to a decrease in the light transmission of the polymer (T). The sample becomes opaque when the 1-butanol content is 20 wt %.

**Figure 12:**
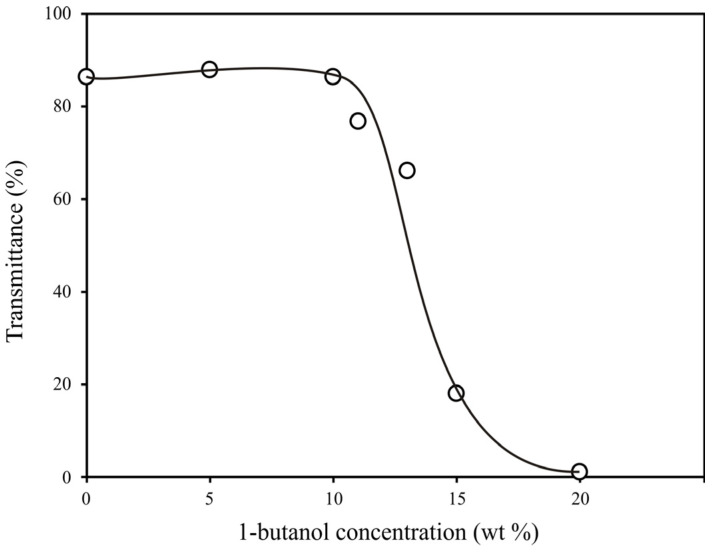
Light transmission of porous polyOCM-2 monoliths at λ = 550 nm depending on the concentration of 1-butanol in the initial PPC.

As seen in Figure 4b, pores ~100 nm in size are present in the polymer obtained from a composition with an alcohol content of 12 wt %. The number and size of large pores increase with an increasing alcohol concentration. This significantly reduces the light transmission of the polymer monolith. The dependence of the light transmission of polymers at λ = 550 nm on the concentration of 1-butanol is shown in Figure 12. It can be seen that this dependence is a stepwise and active formation of pore size in the order of hundreds of microns and occurs at a concentration of 1-butanol in PPC from 10 to 20 wt %.

Thus, changing the light transmission of polyOCM-2 porous monoliths containing 10 wt % 1-butanol and more is caused by an increase in the pore size and is not associated with a change in the structure of the pore system in the polymer. These results are consistent with SEM data (Figure 4).

In the process of composition layer photopolymerization when moving at a certain speed of light and shadow border due to the diffusion of the porogen, it is possible to create a local increase in porogen concentration. It can be smooth. In this case, at low concentrations of the porogen, the polymer layer remains transparent and a gradient of the refractive index is created in it [34]. When a larger amount of the porogen is localized, this can lead to the formation of larger pores in a given area of the layer of the photopolymerizable composition, and this layer becomes light-scattering [35].

### 4.4. Strength Characteristics of polyOCM-2 Porous Monoliths

**Figure 13:**
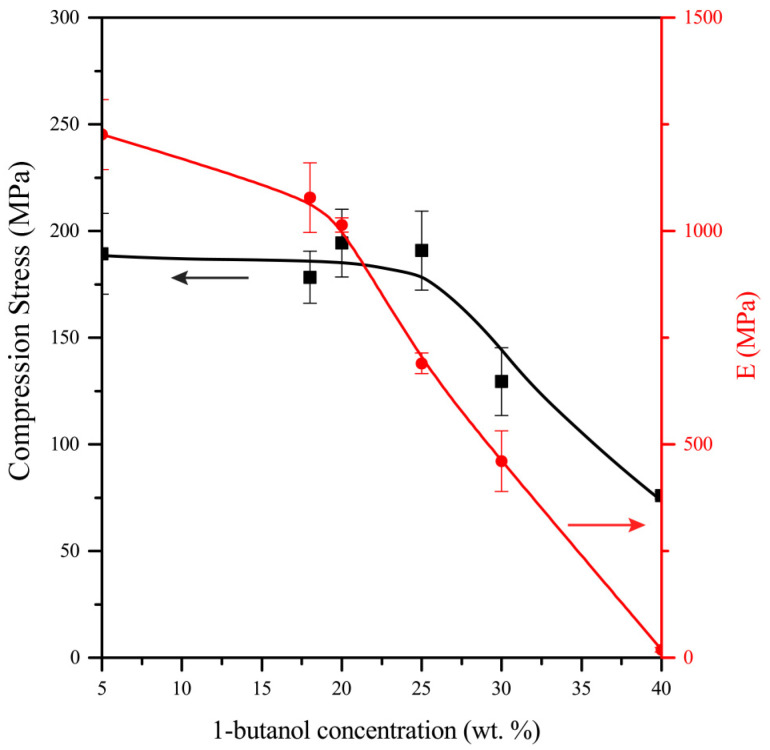
Dependence of the compressive elastic modulus (E_C_) and compressive breaking strength (σ_B_) of polyOCM-2 samples on the concentration of 1-butanol.

One of the most important characteristics of polymers is their strength properties. Figure 13 shows the dependences of the compressive elastic modulus (E_C_) and compressive breaking strength (σ_B_) during compression of polymer samples on the concentration of 1-butanol in the initial photopolymerizable composition. The concentration of 1-butanol varied from 5 to 40 wt %. The dependence of the compressive elastic modulus on the 1-butanol concentration is a broken curve with a break point at a 1-butanol concentration of 20 wt %. The dependence of the compressive breaking strength on the 1-butanol concentration has an expressed stepwise character. A sharp decrease σ_B_ value is observed when the content of 1-butanol in the composition is more than 25 wt %. Thus, changing the type of pore structure from hole-type to interparticle-type when the alcohol content in the composition is more than 20 wt % leads to a decrease in the strength characteristics of the polymer.

## 5. Conclusions

The study of porous monoliths obtained by photopolymerization of OCM-2 dimethacrylate, which forms a crosslinked polymer in the presence of 1-butanol as a porogenic agent, showed that the structure and pore size can be effectively controlled by changing the concentration of the porogenic agent in the initial composition. It should be noted that photopolymerization with visible irradiation makes it possible to eliminate the effect of temperature on the process and to obtain homogeneous polymeric porous monoliths of 2-mm thickness, which ensure high reproducibility and reliability of the results of polymer studies. Depending on the concentration of the porogen in polyOCM-2, two types of porous structures are formed: (i) a hole-type and (ii) an interparticle-type pore structure. The first type includes both open and closed pores; it is formed at a concentration of the porogenic agent up to 20 wt %. Almost entirely consisting of open interconnected pores, the second type structure is formed at a content of more than 30 wt %. For the transition values of porogen concentrations in the polymer, both types of structures are formed. Moreover, the type of porous structure in the bulk of the polymer determines the strength properties of the porous monolith, which change in a step-wise manner when passing from one structure to another. Experimental data indicate the existence of a percolation threshold in the vicinity of a porogenic agent concentration of 15 vol %.

The importance of these studies is determined by the possibility of using biocompatible non-cytotoxic porous polymers based on polyOCM-2 in materials for osteoplasty, which was shown by us earlier [23,24]. Demonstrated in the work, the possibility of obtaining a material with pores of different sizes provides conditions for effective interaction of them with various cell populations. Understanding the principles of obtaining polymeric materials with optimal values of porosity, pore size and strength properties is the most important condition for achieving success in the development of materials for scaffolds.

## Figures and Tables

**Table 1:** Properties of porous poly-OCM-2 monoliths synthesized using various concentrations of porogenic 1-butanol.

Monolith	BuOH, wt %	BuOH, vol %	Dmod, nm	ε, %	Δε, % ^1^	Ssp, m^2^/g	V Total, cm^3^/g
M1	0	0	-	-	-	-	-
M2	10	14.0	3.7	6.4	7.6	19.8	0.05
M3	12	16.9	4.1	6.2	10.7	22.2	0.05
M4	14	19.6	6.2	7.5	12.1	24.7	0.06
M5	16	22.2	12.7	16.0	6.2	49.8	0.14
M6	18	24.7	13.3	15.3	9.4	47.7	0.14
M7	20	27.0	19.7	19.0	8	46.3	0.18
M8	25	33.3	53.2	32.1	1.2	36.3	0.34
M9	30	39.0	255	37.6	1.4	18.8	0.45
M10	35	44.6	715	44.2	0.4	16.6	0.60
M11	40	49.9	927	53.1	−3.2	16.6	0.80
M12	50	59.9	980	59.1	0.8	15.9	1.11
M13	60	69.2	940	69.2	0	16.0	1.64
M14	70	77.7	770	78.4	−0.7	22.2	2.22

^1^ Δ—the difference between the volume concentration of 1-butanol in the composition and the porosity of the polymer.

## Data Availability

The raw/processed data required to reproduce these findings cannot be shared at this time as the data also form part of an ongoing study.
